# Impact of non-uniform attenuation correction in a dynamic [^18^F]-FDOPA brain PET/MRI study

**DOI:** 10.1186/s13550-019-0547-0

**Published:** 2019-08-19

**Authors:** Jorge Cabello, Mihai Avram, Felix Brandl, Mona Mustafa, Martin Scherr, Claudia Leucht, Stefan Leucht, Christian Sorg, Sibylle I. Ziegler

**Affiliations:** 1 Nuklearmedizinische Klinik und Poliklinik, Klinikum rechts der Isar, Technische Universität München, Munich, Germany; 2 Neuroradiology, Klinikum rechts der Isar, Technische Universität München, Munich, Germany; 3 Neuroimaging Center (TUM-NIC), Klinikum rechts der Isar, Technische Universität München, Munich, Germany; 4 Klinik und Poliklinik für Psychiatrie, Klinikum rechts der Isar, Technische Universität München, Munich, Germany; 5 Universitätsklinik für Psychiatrie und Psychotherapie, Paracelsus Medical University, Salzburg, Austria; 6 Klinik und Poliklinik für Nuklearmedizin, Klinikum der Universität München, Ludwig-Maximilians-Universität, Munich, Germany; 7 Present Address: Siemens Healthineers Molecular Imaging, Knoxville, TN USA

**Keywords:** PET/MRI, MR-based attenuation correction, [^18^F]-FDOPA-PET, Patlak, Simplified reference tissue model

## Abstract

**Background:**

PET (positron emission tomography) biokinetic modelling relies on accurate quantitative data. One of the main corrections required in PET imaging to obtain high quantitative accuracy is tissue attenuation correction (AC). Incorrect non-uniform PET-AC may result in local bias in the emission images, and thus in relative activity distributions and time activity curves for different regions. MRI (magnetic resonance imaging)-based AC is an active area of research in PET/MRI neuroimaging, where several groups developed in the last few years different methods to calculate accurate attenuation (μ-)maps. Some AC methods have been evaluated for different PET radioisotopes and pathologies. However, AC in PET/MRI has scantly been investigated in dynamic PET studies where the aim is to get quantitative kinetic parameters, rather than semi-quantitative parameters from static PET studies. In this work, we investigated the impact of AC accuracy in PET image absolute quantification and, more importantly, in the slope of the Patlak analysis based on the simplified reference tissue model, from a dynamic [^18^F]-fluorodopa (FDOPA) PET/MRI study. In the study, we considered the two AC methods provided by the vendor and an in-house AC method based on the dual ultrashort time echo MRI sequence, using as reference a multi-atlas-based AC method based on a T1-weighted MRI sequence.

**Results:**

Non-uniform bias in absolute PET quantification across the brain, from − 20% near the skull to − 10% in the central region, was observed using the two vendor’s μ-maps. The AC method developed in-house showed a − 5% and 1% bias, respectively. Our study resulted in a 5–9% overestimation of the PET kinetic parameters with the vendor-provided μ-maps, while our in-house-developed AC method showed < 2% overestimation compared to the atlas-based AC method, using the cerebellar cortex as reference region. The overestimation obtained using the occipital pole as reference region resulted in a 7–10% with the vendor-provided μ-maps, while our in-house-developed AC method showed < 6% overestimation.

**Conclusions:**

PET kinetic analyses based on a reference region are especially sensitive to the non-uniform bias in PET quantification from AC inaccuracies in brain PET/MRI. Depending on the position of the reference region and the bias with respect to the analysed region, kinetic analyses suffer different levels of bias. Considering bone in the μ-map can potentially result in larger errors, compared to the absence of bone, when non-uniformities in PET quantification are introduced.

## Introduction

PET/MRI (positron emission tomography/magnetic resonance imaging) represents a comprehensive tool for neurology and neuro-oncology studies. Different works have illustrated the complementary information provided by PET/MRI imaging, allowing to better understand the underlying pathophysiological mechanisms of numerous pathologies [1, 2]. However, the understanding of the information provided by PET/MRI technology represents a challenge in some scenarios.

One of the technical challenges posed by PET/MRI is to extract an attenuation map (μ-map), for PET attenuation correction (AC), that accurately resembles the underlying electron density distribution of the subject under study. Since MRI provides proton density information, an intermediate step is required to convert proton density information to electron density, in the form of linear attenuation coefficients (LAC) at the PET energy of 511 keV. The two-point Dixon MRI sequence was initially proposed to identify air, fat, lung, and soft tissue, which has been demonstrated to provide high quantitative accuracy for whole-body PET/MRI [3]. However, the case of brain PET/MRI AC is more challenging, due to the high-density bone located in the head and the large solid angle coverage of the bone.

Since the first PET/MRI scanners were installed, early studies already showed the low accuracy obtained with the Dixon-based μ-map for PET reconstruction in neurological studies, indicating the importance of including bone in the μ-map [4]. Since the problem of AC in PET/MRI for neurological studies was first demonstrated, numerous methods based on a variety of approaches were developed by different groups. The first methods were based on segmenting MRI sequences that provided bone information (dual ultrashort time echo—dUTE), with the aim of identifying bone, soft tissue, and air [5], and further developments [6–8]. Other methods employed matched databases containing computed tomography (CT) and MRI data [9, 10] or templates based on the same concept [11, 12]. More recently, matching CT/MRI data was also exploited using deep learning approaches, producing accurate results with a small training dataset [13]. All these studies evaluated a novel method to calculate a μ-map and compared with the methods provided by the vendor using CT as ground truth. Additional comparative studies investigated the accuracy of several of the newly developed methods, compared to the approaches from the vendor, using CT as reference [14, 15]. Alzheimer patients with [^18^F]-fludeoxyglucose (FDG), [^11^C]-Pittsburgh compound B (PiB), and [^18^F]-florbetapir were included in [15], concluding that all methods performed in a < 5% error compared to CT.

Most studies, devoted to evaluating the accuracy of AC in PET/MRI, were focused in neuro-oncological [16], neurological [6, 7, 9], or healthy volunteers [11], for static PET studies. From the initial studies, it was concluded that PET quantification errors due to inaccurate μ-maps were highly non-uniform, ranging from 5–10% for areas in the central region of the brain to 25% for regions close to bone [4]. This finding was especially relevant for PET studies where the data are normalised to a reference region [17], and PET dynamic studies based on an input function extracted from a reference tissue [18].

Works evaluating the impact of AC in dynamic PET studies are scant. Exemplary, the work by Mérida et al. compared the effects of several atlas-based in-house-developed μ-maps, on static [^18^F]-FDG-PET and dynamic [^18^F]-2′-methoxyphenyl-(*N*-2′-pyridinyl)-p-fluoro-benzamidoethyipiperazine (MPPF)-PET analysis [10]. [^18^F]-MPPF-PET is used to target the 5-HT 1 A receptor, to study serotonergic disorders associated with neuropsychiatric problems. [^18^F]-MPPF has been analysed using compartmental modelling based on the simplified reference tissue model (SRTM), using the cerebellum as reference region [19]. Results revealed position-dependent bias of non-displaceable binding potential (BP_ND_) with respect to the distance with bone in the analysed regions: hippocampus, and anterior and posterior temporal lobe.

Another recent work evaluated a new method to calculate the μ-map based on neural networks compared to transmission scans, on dynamic [^11^C]-WAY-100635- and [^11^C]-DASB-PET studies [20]. These PET radiotracers are employed to target 5-HT 1A receptors and 5-HTT serotonin transporter, respectively. The kinetic models used were a two-tissue compartment model (2TCM) using the cerebellar white matter as reference region and graphical analysis method, respectively. Results concluded that differences between both μ-maps were below the test-retest variability measured in previous studies.

Focusing more on the impact of AC in the kinetic model, Mansur and colleagues evaluated the impact of an atlas-based MRI-based AC [9] in dynamic [^11^C]-Cimbi-36-PET kinetic analysis, compared to CT-based AC [21]. [^11^C]-Cimbi-36 is a PET radioligand designed to target 5-HT 2A receptors, associated with a wide range of neurological diseases. Two kinetic models were evaluated: one based on SRTM (cerebellum) and a 2TCM using an arterial plasma input function. Results indicated that the differences in BP_ND_ values measured with both μ-maps and both kinetic models were not statistically significant. However, higher BP_ND_ values were consistently measured with the SRTM than with the 2TCM in the amygdala, but not in the hippocampus.

Lassen and colleagues explored the discrepancies found when transferring static and dynamic PET-only protocols to a PET/MRI system [22]. The dynamic studies were focused on (R)-[ ^11^C]verapamil, a tracer used to study the P-glycoprotein present in the blood-brain barrier, suspected to be related to several neurodegenerative disorders. One-tissue compartmental modelling using arterial blood sampling was implemented. The evaluated μ-maps were a Dixon-based from the PET/MRI system and a CT-based, using a transmission-based μ-map from the PET-only system as reference. Underestimations between − 36 and 5% were found for the different kinetic parameters in the PET/MRI using the Dixon-based μ-map, while these underestimations decreased to − 17–3% using the CT-based μ-map in the different analysed regions: insula and parietal lobe, as well as in the whole brain.

The goal of this work is to study the influence of AC in a dynamic brain [^18^F]-FDOPA-PET/MRI study. The suitability of [^18^F]-FDOPA-PET to identify neurological disorders, where the dopaminergic system is involved, has been investigated in several studies on schizophrenia [23] and Parkinson’s disease [24]. Different approaches have been employed to analyse the kinetics of [^18^F]-FDOPA-PET, from micro-kinetic modelling using two-tissue compartmental analysis, employing arterial sampling as input function [25], to macro-kinetic modelling using linearised models (Patlak analysis) based on SRTM [23, 26]. It is important to note that the latter is being more adopted in clinical setups due to its simplicity and low complexity in scan protocol and patient preparation. The target of these analyses was generally the striatum and the subregions of the striatum, which is where the highest density of dopaminergic synapses is found.

We performed for the first time a simultaneous brain [^18^F]-FDOPA-PET/MRI study with the Biograph mMR scanner (Siemens Healthineers AG, Erlangen, Germany). Previous brain [^18^F]-FDOPA-PET studies were performed in PET systems, followed usually by an MRI scan to delineate the anatomical regions, especially the striatum and subregions of the striatum. AC in these previous studies was performed using CT from the PET/CT [26, 27] or a rotating transmission source [23, 28, 29]. As aforementioned, and as indicated by other studies [22], translating PET-only studies to PET/MRI scanners requires careful evaluation and assessment regarding AC.

In this work, we evaluated the accuracy of the different μ-maps provided by the vendor and an in-house-developed μ-map derived from the dUTE-MRI sequence, which is always acquired in all our brain PET studies [6]. Due to the absence of a CT scan from the subjects included in this work, we compared with an atlas-based μ-map derived from the magnetization-prepared rapid gradient-echo (MP-RAGE)-MRI sequence [9], which has shown to produce errors of < 2% in quantification accuracy in different brain regions [16], being lower than the test-retest error of < 6% measured in the striatum with [^18^F]-FDOPA [30]. It is worth highlighting that the MP-RAGE sequence is not always acquired in our clinical brain PET/MRI protocols due to its long duration (5 min). The comparison between μ-maps was evaluated at different levels: amount and position of bone tissue, absolute activity concentration accuracy, and of calculated kinetic parameters. The novelty of the present work lies in the study of the influence of non-uniform AC accuracy in a dynamic brain [^18^F]-FDOPA-PET study, where a reference region is used for the kinetic analysis.

## Materials and methods

### Patient population, protocol, and reconstruction

Twenty-five healthy controls and 26 schizophrenic patients were scanned for this prospective study in the Biograph mMR (VB20P). The subjects were injected a bolus of 140 ± 21 MBq of [^18^F]-FDOPA 30 s after the start of the dynamic PET scan, which lasted 70 min. The PET scan was acquired together with a series of MRI sequences, including a 2-point Dixon, a dUTE, and a T1w three-dimension magnetization-prepared rapid gradient-echo (MP-RAGE). The parameters of the 2-point Dixon were repetition-time/echo-time/flip-angle of 3.6 ms/2.26 ms/10°, and a field of view 449 × 295 × 349 mm^3^, with a voxel size of 2.34 × 2.34 × 2.73 mm^3^. The dUTE consisted of two ultrashort echo time sequences at consecutive echo-times (0.07 ms and 2.36 ms), repetition-time/flip-angle of 3.98 ms/10°, and a field of view 300 × 300 × 300 mm^3^ with an isotropic voxel size of 1.56 mm. The parameters of the MP-RAGE were repetition-time/echo-time/flip-angle of 2.3 ms/2.98 ms/9°, and a field of view 256 × 256 × 160 mm^3^ with an isotropic voxel size of 1.0 mm.

Data from each PET scan were acquired in list-mode format and organised in a 30-s blank frame, followed by 10 × 15 s, 3 × 20 s, 2x60 s, 2 × 120 s, and 12 × 300 s frames. Each frame was reconstructed using the ordered subsets expectation maximisation algorithm, with 24 subsets and 3 iterations, and filtered with a 3-mm full-width at half-maximum Gaussian filter. The voxel size was 1.66 × 1.66 × 2.03 mm^3^ in a field of view of 359 × 359 × 258 mm^3^. Reconstruction was performed using an offline reconstruction toolkit provided and validated by the vendor, which includes attenuation, scatter and decay corrections, and normalisation.

### Attenuation maps

The vendor provides two different μ-maps in the VB20P version of the scanner software: one based on the Dixon sequence (μ-map_DXN_) and another one based on the dUTE sequence (μ-map_dUTE_). The former includes soft tissue (LAC 0.1 cm^−1^), fat (LAC 0.0854 cm^−1^), and air, while the latter also includes bone (LAC 0.15 cm^−1^). Additionally, to the two μ-maps from the vendor, we also calculated another μ-map based on segmenting the R_2_-map obtained from the dUTE sequence [6] (μ-map_dUTE−R2_), and a multi-atlas-based μ-map obtained from the MP-RAGE sequence [9], referred as pseudo-CT (pCT) in the following. The method based on the R_2_-map relies on two UTE images consecutively acquired at two different echo-times: 0.07 ms and 2.36 ms. After correcting the bias field in these two UTE images using Statistical Parametric Mapping-12 (SPM^1^), the R_2_-map is calculated as:

1 $$ {R}_2=\frac{\log {I}_1-\log {I}_0}{{\mathrm{TE}}_1-{\mathrm{TE}}_0}, $$

where *I*_0_ and *I*_1_ are the resulting images from the two consecutive echo images at TE_0_ (2.36 ms) and TE_1_ (0.07 ms), respectively [5]. Air cavities are extracted from the two UTE images, and bone is extracted from the R_2_-map, using in both cases adaptive thresholding. Continuous LACs are assigned to the bone by scaling the intensity values from the R_2_-map to a database of double-scanned patients with dUTE and CT data, acquired in a previous study [6].

The method based on the atlas derives the μ-map from the patient MP-RAGE sequence. A database of MP-RAGE images and a matched database of CT images are used to match the patient-specific MP-RAGE sequence using linear and non-linear transformations. The patient-specific MP-RAGE images are normalised to the MP-RAGE template, followed by an inverse normalisation applied to the CT template [9]^2^.

### Data workflow

After all the frames were reconstructed, rigid motion correction, using SPM, was applied to every frame, using the PET frame at 35 min post-injection as reference, concurring with the MP-RAGE acquisition.

Subsequently, the masks of the regions of interest for the kinetic analysis were automatically calculated. These regions were the cerebellar cortex and the occipital pole (for the Patlak-SRTM analysis), the striatum, and the striatal subregions based on the anatomical classification: caudate, putamen, and accumbens, extracted from the Harvard-Oxford subcortical atlas [31]. The procedure is briefly described below:

1. SPM internally resamples the MP-RAGE data (1.0 × 1.0 × 1.0 mm^3^) to the PET data space (1.7 × 1.7 × 2.0 mm^3^).
2. Resampled MP-RAGE is normalised to SPM brain template in MNI space.
3. Inverse normalisation is applied to pre-selected individual brain regions in MNI space (cerebellar cortex, occipital pole, striatum, and subregions from striatum) to convert them to PET space.

### Figures of merit

The correlation between the bone distribution in the μ-maps was measured with the Jaccard index and the normalised mutual information (NMI). The Jaccard index can be defined as:

2 $$ J\left({\mu}_1,{\mu}_2\right)=\frac{\left|{\mu}_1\cap {\mu}_2\right|}{\left|{\mu}_1\cup {\mu}_2\right|}, $$

where *μ*_1_ and *μ*_2_ are the two μ-maps being evaluated. For the Jaccard distance, the μ-maps were binarised using a threshold of 0.109 cm^−1^.

The NMI is defined as:

3 $$ \mathrm{NMI}\left({\mu}_1,{\mu}_2\right)=\frac{H\left({\mu}_1\right)+H\left({\mu}_2\right)}{H\left({\mu}_1,{\mu}_2\right)}, $$

where *H*(*μ*) is the entropy of *μ*, measured as:

4 $$ H\left(\mu \right)={\sum}_{i=1}^np\left({\mu}_i\right){\log}_2\left[p\left({\mu}_i\right)\right], $$

where *i* is the voxel index, *n* the total number of voxels in the intersection of the μ-maps, and *p(μ*_*i*_*)* the probability of occurrence *μ*_*i*_, usually calculated as the histogram of *μ*. *H*(*μ*_1_, *μ*_2_) is the joint entropy between *μ*_1_ and *μ*_2_, measured as:

5 $$ H\left({\mu}_1,{\mu}_2\right)={\sum}_{i=1}^n{\sum}_{j=1}^np\left({\mu}_{1i},{\mu}_{2j}\right){\log}_2\left[p\left({\mu}_{1i},{\mu}_{2j}\right)\right] $$

where *p(μ*_1*i*_*, μ*_2*j*_*)* is the joint probability of *μ*_1*i*_ and *μ*_2*j*_ occurring together. Activity concentrations measured in PET images were evaluated by calculating the normalised error (E_n_), defined as:

6 $$ {E}_n=100\frac{A_c-{A}_{c, ref}}{A_{c, ref}} $$

where *A*_*c*_ is the activity concentration measured in a PET image, using the reconstructed PET images obtained with the pCT as reference (*A*_*c,ref*_). The averaged E_n_ was calculated in the striatum, cerebellar cortex, and occipital pole, the ROIs under evaluation for this study. Moreover, to investigate the intra-region variations, we also calculated a voxel-wise E_n_ brain map using the following procedure:

1. The last PET frame (65–70 min), reconstructed using the pCT as μ-map, from each subject was normalised to the SPM brain template in MNI (Montreal Neurological Institute) space.
  The PET reconstructed images obtained with the other μ-maps (μ-map DXN, μ-map_dUTE_, μ-map_dUTE−R2_) were normalised to the SPM brain template using the same normalisation calculated in the previous step.
2. The E_n_ per voxel from each subject for each evaluated μ-map was calculated (in MNI space) using the PET reconstructed image obtained using pCT as reference.
3. The averaged E_n_ map was calculated by averaging the E_n_ obtained for each subject.

### Kinetic analysis

In this study, we used the Gjedde-Patlak graphical analysis [32] based on the SRTM [18], defined as:

7 $$ \frac{C_s(t)}{C_c(t)}={K}_i\frac{\int_0^t{C}_c(t)}{C_c(t)}+{V}_0, $$

where *C*_*s*_(*t*) and *C*_*c*_(*t*) are the time activity curve (TAC) measured in any given voxel in the PET image and the TAC measured in a reference tissue region, respectively. *V*_0_ and *K*_*i*_ represent the initial volume of distribution and the maximum capacity of dopamine synthesis, respectively. This model was employed under the assumption that the [^18^F]-FDOPA kinetic model behaves irreversible, which has been confirmed in previous studies for scans shorter than 70 min [33]. The slope from the Patlak plot (*K*_*i*_), calculated between 20–60 min, was used as a macro-kinetic parameter describing the dopamine synthesis capacity. We evaluated two different reference regions considered in previous studies: the cerebellar cortex—*K*_*i*_^cer^ [23]—and the occipital pole—*K*_*i*_^op^ [28]. Both regions are located close to the bone, and the non-uniform attenuation effect can potentially affect differently the kinetic analysis, depending on which region is used as reference. *K*_*i*_ was measured in the whole striatum and in the subregions of the striatum: caudate, nucleus accumbens, and putamen, extracted from the Harvard-Oxford subcortical atlas [34].

Differences between the measured macro-kinetic parameter *K*_*i*_ across subjects using each μ-map were statistically evaluated using a repeated measures ANOVA followed by post hoc tests between each μ-map. The statistical analysis was performed for both: *K*_*i*_^cer^ and *K*_*i*_^op^. We used SPSS (IBM Corp. Released 2013. IBM SPSS Statistics for Windows, version 22.0. Armonk, NY: IBM Corp.) for the statistical analysis.

## Results

### Bone identification accuracy

**Fig. 1:**
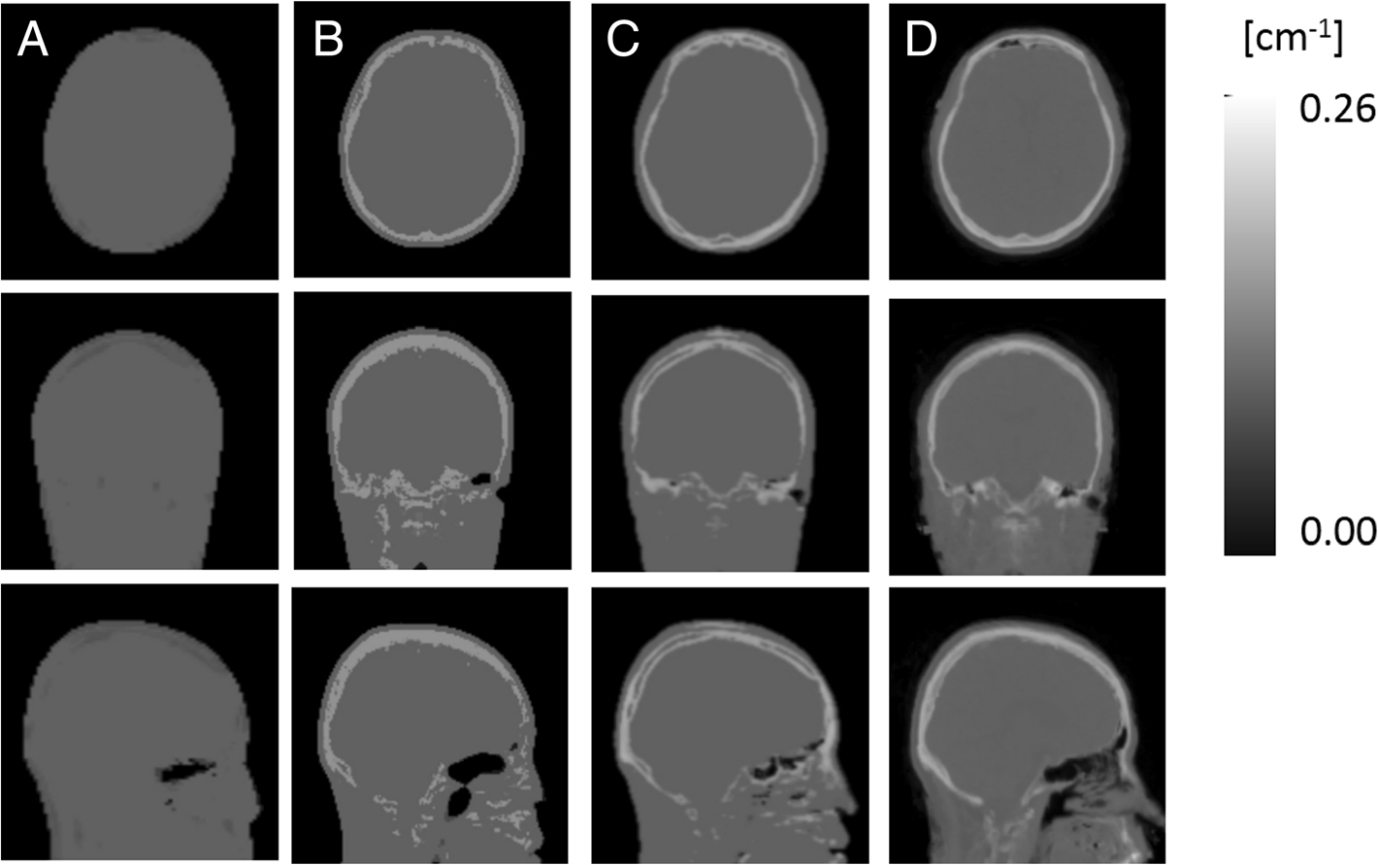
Axial (top), coronal (middle), and sagittal (bottom) views of an exemplar μ-map_DXN_ (**A**), μ-map_dUTE_ (**B**), μ-map_dUTE−R2_ (**C**), and pCT (**D**)

Figure 1 shows the μ-map_DXN_, μ-map_dUTE_, μ-map_dUTE−R2_, and pCT from one exemplar subject. As aforementioned, the μ-map_DXN_ does not identify bone, while the μ-map_dUTE_ from the vendor uses one single LAC to the entire bone (0.15 cm^−1^), and the other two μ-maps contain continuous LAC values in the bone.

The overlapping of bone between the different μ-maps was measured with the Jaccard distance and NMI. Table 1 shows the Jaccard index and NMI obtained with μ-map_dUTE_ and μ-map_dUTE−R2_ compared to pCT. For both figures of merit, μ-map_dUTE−R2_ outperformed μ-map_dUTE_.

**Table 1:** Bone overlapping indices: the Jaccard index and NMI between μ-map_dUTE_ and μ-map_dUTE−R2_ with the pCT

	Jaccard index	NMI
μ-map_dUTE_	0.39 ± 0.07	0.37 ± 0.06
μ-map_dUTE−R2_	0.53 ± 0.06	0.48 ± 0.06

### PET region of interest analysis—time activity curves

**Fig. 2:**
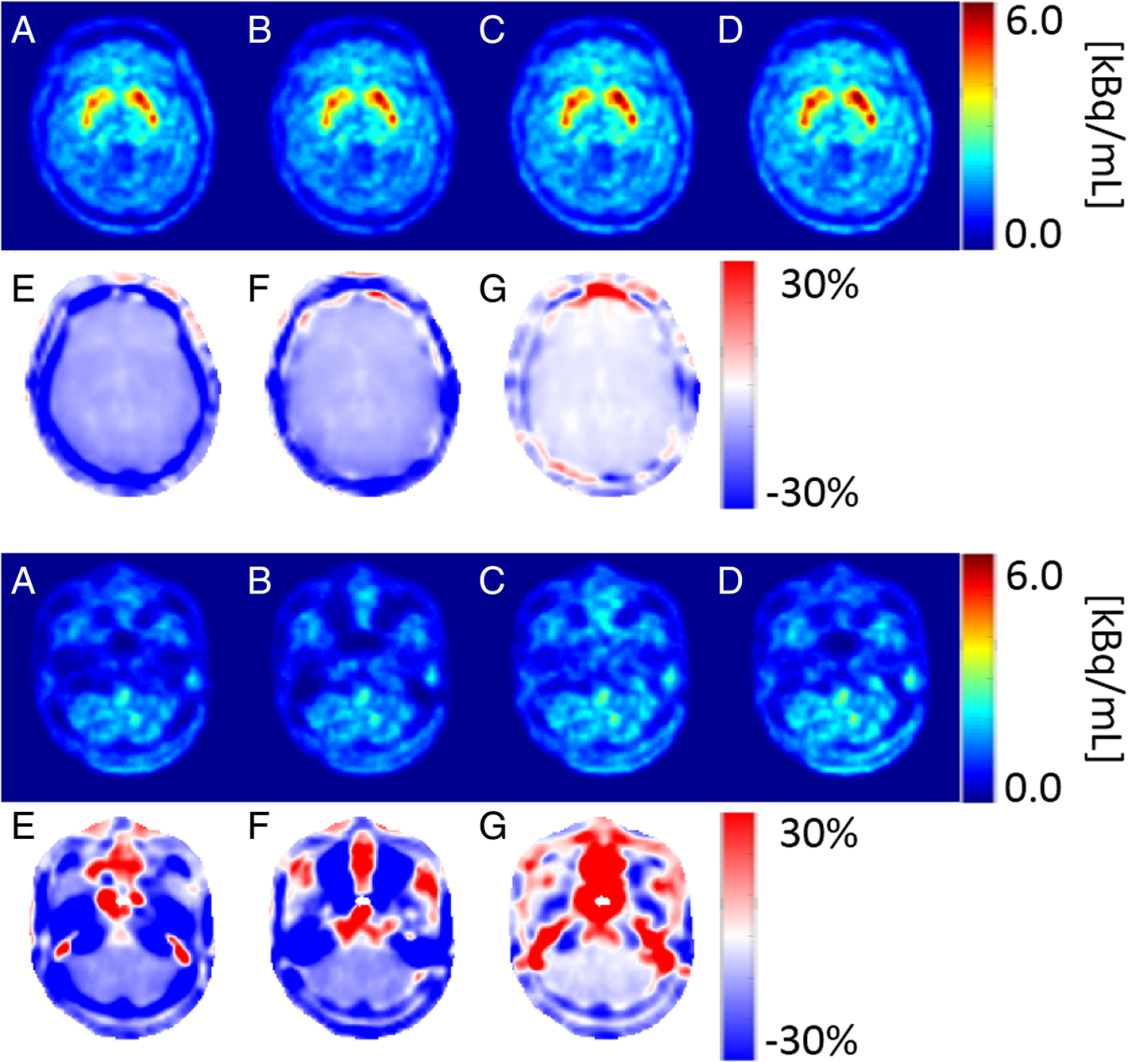
Axial slices of the last PET frame (65–70 min) from one subject at the height of striatum and occipital pole (top), and cerebellum (bottom) reconstructed using μ-map_DXN_ (A), μ-map_dUTE_ (B), μ-map_dUTE−R2_ (C), and pCT (D). En between the PET datasets obtained using μ-map_DXN_ (E), μ-map_dUTE_ (F), and μ-map_dUTE−R2_ (G) with pCT

Figure 2 shows axial slices of the last PET frame (65–70 min) at the height of the striatum, occipital pole, and cerebellar cortex of an exemplar subject, reconstructed using μ-map_DXN_, μ-map_dUTE_, μ-map_dUTE−R2_, and pCT, together with the E_n_ obtained in each case using the pCT as reference. The highest error was observed in regions of bone and air cavities. In contrast, the error measured in the brain was in general lower, in agreement with previous studies [6, 11].

**Fig. 3:**
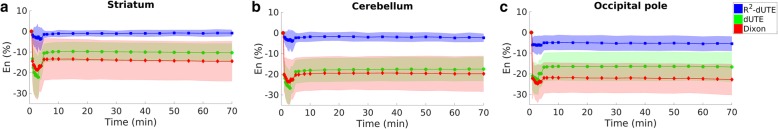
En and standard deviation among subjects obtained with the μ-map_dUTE−R2_ (blue), μ-map_dUTE_ (green), and μ-map_DXN_ (red), measured in the striatum (**a**), cerebellar cortex (**b**), and occipital pole (**c**)

Figure 3 shows the mean E_n_ (solid line) and standard deviation (shaded area) across subjects, measured in absolute PET activity concentration in the striatum, cerebellar cortex, and occipital pole for each time point, obtained with μ-map_DXN_, μ-map_dUTE_, μ-map_dUTE−R2_, and pCT as reference.

The first time points showed high variations due to the low statistics contained in the first frames, but all the analysed regions showed stable bias after 8 min. The mean and standard deviation for the E_n_ measured in the TACs between 8 and 70 min are shown in Table 2, for the striatum, cerebellar cortex, and occipital pole, using μ-map_DXN_, μ-map_dUTE_, and μ-map_dUTE−R2_ for the PET reconstruction.

**Table 2:** Mean E_n_ (%) and mean standard deviation in PET activity concentration across subjects averaged over time (8–70 min)

	Striatum	Cerebellum	Occipital pole
μ-map_DXN_	− 13.94 ± 9.43%	− 19.58 ± 8.12%	− 22.2 ± 7.10%
μ-map_dUTE_	− 9.99 ± 4.36%	− 17.7 ± 5.58%	− 16.4 ± 6.82%
μ-map_dUTE−R2_	− 0.93 ± 1.50%	− 1.98 ± 2.12%	− 5.1 ± 3.51%

The most inaccurate results were consistently obtained with the μ-map_DXN_, followed by the μ-map_dUTE_. In addition, μ-map_DXN_ showed higher variability among subjects compared to the μ-map_dUTE−R2_ for the three analysed regions. The largest differences between AC methods were observed in the occipital pole, demonstrating that this is the most sensitive region to AC inaccuracies. Additionally, the two AC methods that include bone in the μ-map (μ-map_dUTE_ and μ-map_dUTE−R2_) showed the highest variability in the occipital pole, while the μ-map_DXN_ showed the highest variability in the striatum.

**Fig. 4:**
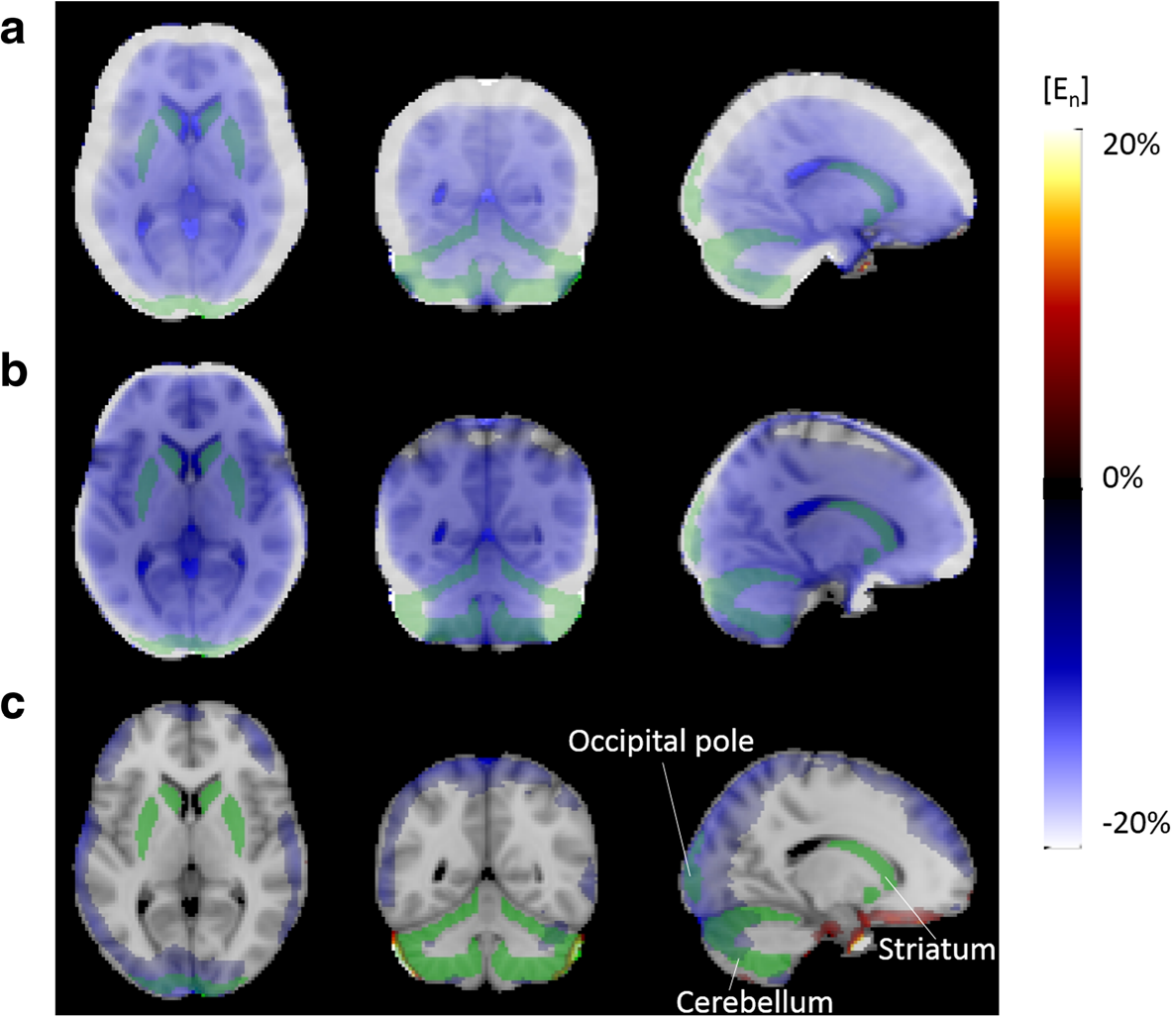
Axial (left), coronal (middle), and sagittal (right) views of the normalised voxel-wise En maps obtained with the μ-map_DXN_ (**a**), μ-map_dUTE_ (**b**), and μ-map_dUTE−R2_ (**c**), overlaid on the MRI template. Striatum, cerebellar cortex, and occipital pole regions are indicated in green

Figure 4 shows the axial, coronal, and sagittal views of the voxel-wise E_n_ map normalised across all subjects, obtained from the PET images reconstructed with each of the μ-maps under evaluation, using pCT as reference. The E_n_ maps are overlaid on the brain MRI template used for the normalisation, together with the striatum, cerebellar cortex, and occipital pole masks in MNI space. E_n_ < 6% are not shown in the E_n_ maps since they are below the test-retest error. The μ-map_DXN_ shows high E_n_ in the entire outer region of the brain, while the μ-map_dUTE_ shows high E_n_ especially in the occipital and frontal regions, and close to the sinus. The μ-map_dUTE−R2_ shows E_n_ < − 6% in focalized areas in the parietal and frontal regions, similar to μ-map_dUTE_ but to a lesser extent, and E_n_ > 6% close to the sinus. Figure 4 also shows the extension of the non-uniformity across the brain, highlighting the importance of the relative location of a reference region with respect to the region of interest.

### PET kinetics evaluation

**Fig. 5:**
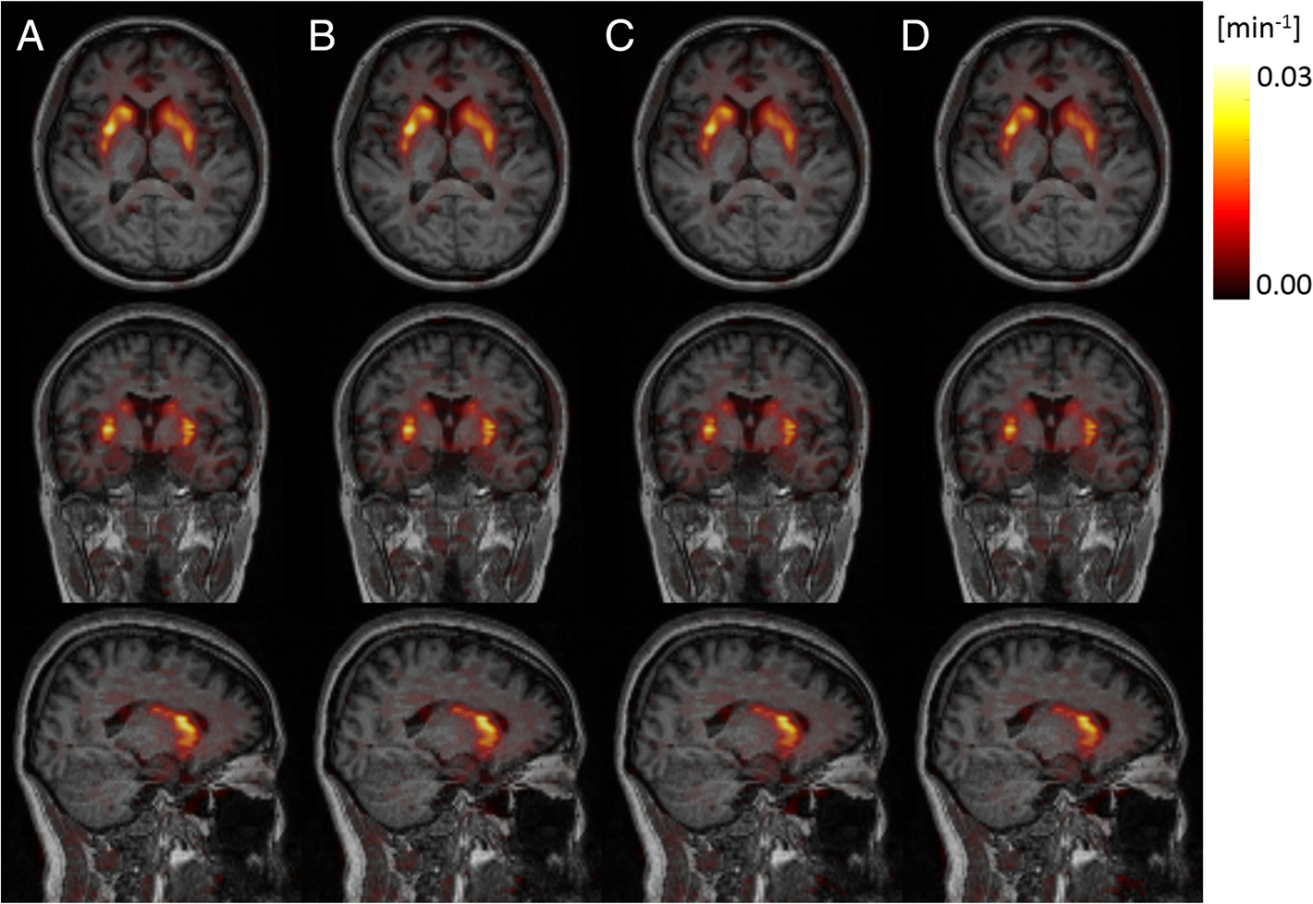
Axial (top), coronal (middle), and sagittal (bottom) views of an exemplar *Ki* parametric maps using the μ-map_DXN_ (**a**), the μ-ma_pdUTE_ (**b**), the μ-map_dUTE−R2_ (**c**), and the pCT (**d**), obtained using the cerebellar cortex as reference region

Figure 5 shows the axial, coronal, and sagittal views of one exemplar *K*_*i*_ parametric map obtained with the μ-map_DXN_, μ-map_dUTE_, μ-map_dUTE−R2_, and pCT, using the cerebellar cortex as reference region. No differences were visually observed between *K*_*i*_ parametric maps using the different μ-maps.

**Fig. 6:**
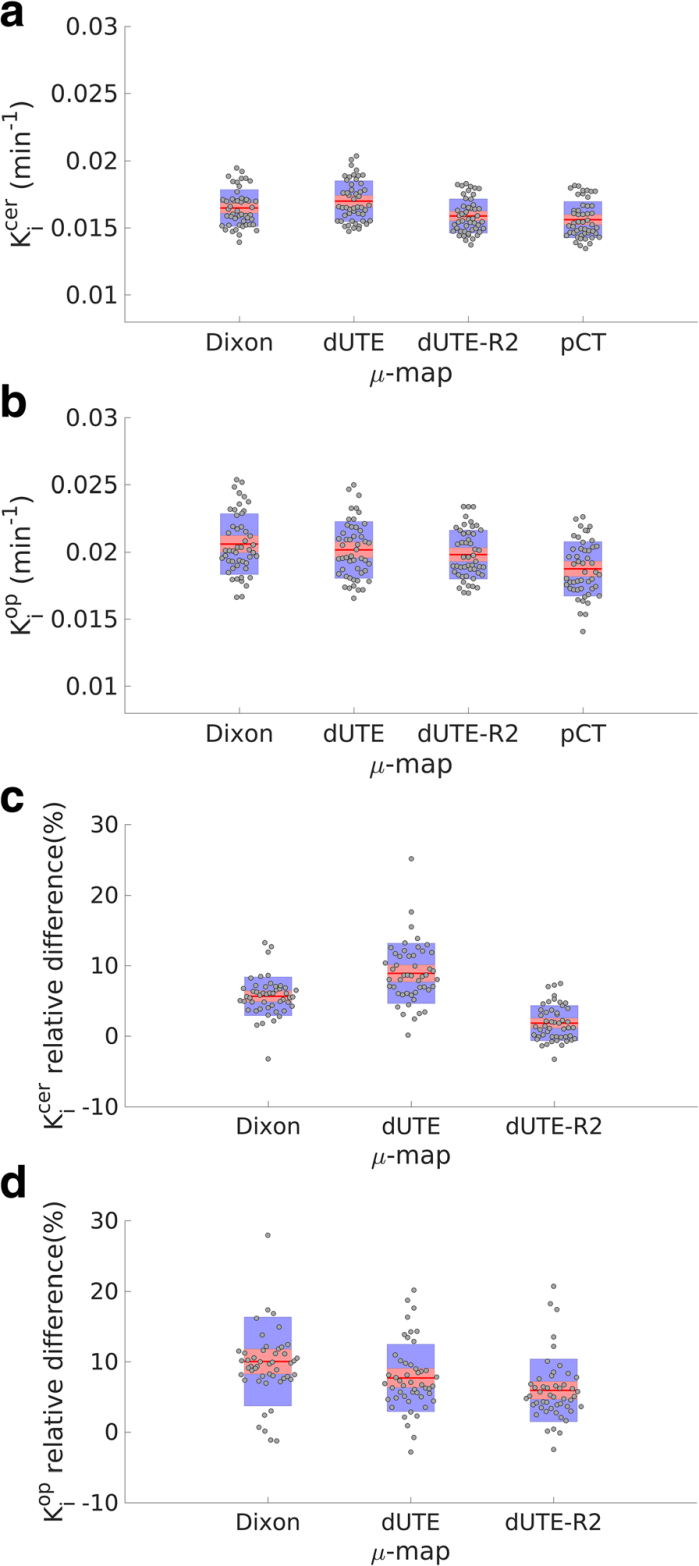
*K*_*i*_^cer^ (**a**) and *K*_*i*_^op^ (**b**) measured in each subject in the region of the striatum obtained with reconstructed PET images calculated with the μ-map_DXN_, μ-map_dUTE_, μ-map_dUTE−R2_, and pCT. The relative differences measured in each subject using the pCT as reference are shown in **c** and **d** for cerebellar cortex and occipital pole, respectively

Figure 6 shows the averaged *K*_*i*_^cer^ (Fig. 6a) and *K*_*i*_^op^ (Fig. 6b) values measured in the striatum, for each subject obtained with the μ-map_DXN_, μ-map_dUTE_, μ-map_dUTE−R2_, and pCT. The boxplot in each case illustrates the mean, the 95% confidence interval, and one standard deviation. Statistically significant differences (*p* < 0.001) in *K*_*i*_^cer^ and in *K*_*i*_^op^ values were measured between all the μ-maps (μ-map_DXN_, μ-map_dUTE_, and μ-map_dUTE−R2_), compared with those obtained with the pCT. The statistically significant differences were measured in the entire striatum and in the subregions of the striatum. The relative difference (E_n_) measured between *K*_*i*_ values for each subject, using the pCT as reference, is shown in Fig. 6c and d for the cerebellar cortex and the occipital pole, respectively.

Figure 6 demonstrates that the *K*_*i*_^op^ values are consistently higher and show higher variability amount subjects than the *K*_*i*_^cer^ values. Interestingly, while using the occipital pole as reference region, the μ-map_DXN_ produces the most inaccurate results. However, using the cerebellar cortex as reference region, the μ-map_dUTE_ showed the most inaccurate results. This effect can be explained by the larger relative differences in E_n_ measured between the striatum and cerebellar cortex with the μ-map_dUTE_, as compared to the smaller relative difference with the μ-map_DXN_ (Table 2). These findings illustrate that μ-map_dUTE_ introduces higher E_n_ non-uniformities, compared to μ-map_DXN_, when using the cerebellar cortex as reference region, even though the global E_n_ is lower.

The averaged *K*_*i*_^cer^ in the striatum and its different sub-regions, obtained with each μ-map using the cerebellar cortex as reference region, is shown in Table 3. Similarly, the averaged *K*_*i*_^op^ in the striatum and its different sub-regions, obtained with each μ-map using the occipital pole as reference region, is shown in Table 4.

**Table 3:** Mean *K*_*i*_^cer^ and standard deviation (cm^−1^)

	μ-map_DXN_	μ-map_dUTE_	μ-map_dUTE−R2_	pCT
Striatum	0.016 ± 0.001	0.016 ± 0.002	0.016 ± 0.001	0.016 ± 0.001
Putamen	0.017 ± 0.002	0.018 ± 0.002	0.017 ± 0.001	0.016 ± 0.001
Caudate	0.015 ± 0.002	0.016 ± 0.002	0.015 ± 0.002	0.014 ± 0.002
Accumbens	0.015 ± 0.002	0.015 ± 0.002	0.014 ± 0.002	0.014 ± 0.002

**Table 4:** Mean *K*_*i*_^op^ and standard deviation (cm^−1^)

	μ-map_DXN_	μ-map_dUTE_	μ-map_dUTE−R2_	pCT
Striatum	0.020 ± 0.001	0.020 ± 0.002	0.020 ± 0.001	0.019 ± 0.001
Putamen	0.021 ± 0.002	0.021 ± 0.002	0.021 ± 0.001	0.020 ± 0.001
Caudate	0.019 ± 0.002	0.018 ± 0.002	0.018 ± 0.002	0.017 ± 0.002
Accumbens	0.019 ± 0.002	0.018 ± 0.002	0.018 ± 0.002	0.017 ± 0.002

The mean (and standard deviation) relative difference measured in the *K*_*i*_^cer^ in the striatum and its subregions compared to the *K*_*i*_^cer^ obtained with the pCT is shown in Tables 5 and 6, for the cerebellar cortex and occipital pole, respectively.

**Table 5:** Mean and standard deviation of the relative differences of *K*_*i*_^cer^ (%)

	μ-map_DXN_	μ-map_dUTE_	μ-map_dUTE−R2_
Striatum	5.64 ± 2.74	8.90 ± 4.27	1.83 ± 2.49
Putamen	5.57 ± 2.67	8.55 ± 4.01	1.76 ± 2.52
Caudate	5.59 ± 3.08	9.72 ± 4.45	1.87 ± 2.57
Accumbens	5.85 ± 2.87	8.84 ± 8.27	1.84 ± 2.79

**Table 6:** Mean and standard deviation of the relative differences of *K*_*i*_^op^ (%)

	μ-map_DXN_	μ-map_dUTE_	μ-map_dUTE−R2_
Striatum	10.03 ± 6.31	7.69 ± 4.78	5.93 ± 4.45
Putamen	9.95 ± 6.57	7.33 ± 4.92	5.87 ± 4.66
Caudate	9.90 ± 5.95	8.44 ± 5.00	5.94 ± 4.18
Accumbens	10.25 ± 6.15	7.42 ± 7.06	5.96 ± 4.41

**Fig. 7:**
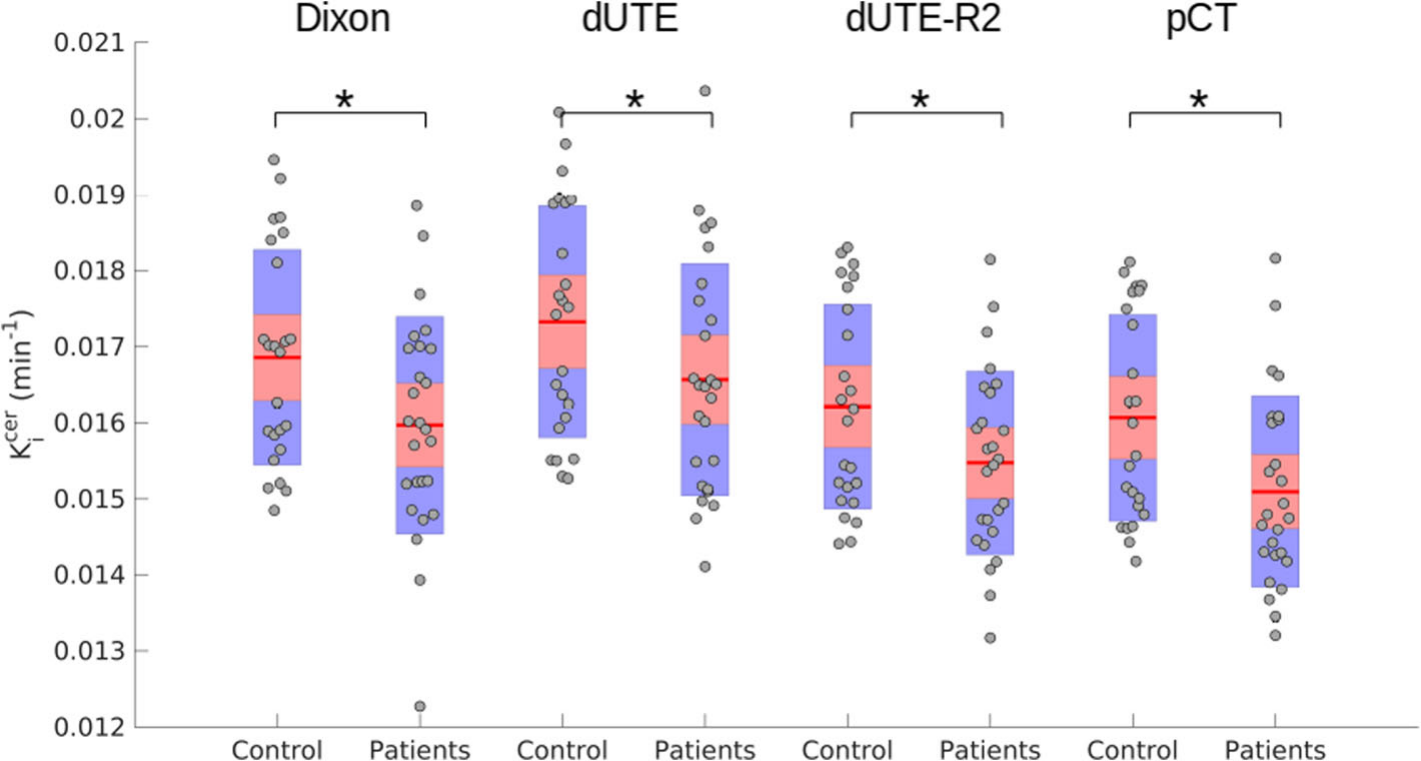
*K*_*i*_^cer^ values measured in the striatum region for each subject using all the μ-maps, separated by healthy controls and patients. The statistical significance measured between groups for each μ-map is shown as “*” for *p* < 0.05

To demonstrate the clinical impact of the different levels of attenuation correction accuracy, we studied the differential signal in *K*_*i*_ values measured between healthy controls and patients. Figure 7 shows the resulting *K*_*i*_^cer^ values per subject, separated in healthy controls and patients, showing the statistical significance (evaluated by a two-sample *t* test) between groups for each μ-map. Table 7 shows the mean *K*_*i*_^cer^ values per group and their *p* value. No statistically significant difference was measured between groups in the *K*_*i*_^op^ values.

**Table 7:** Mean *K*_*i*_^cer^, standard deviation (min^−1^), and *p* value between healthy controls and patients

	Healthy controls	Patients	*p* value
μ-map_DXN_	0.0169–0.001	0.0169–0.001	0.018
μ-map_dUTE_	0.0173–0.001	0.0166–0.001	0.031
μ-map_dUTE−R2_	0.0162–0.001	0.0155–0.001	0.022
pCT	0.0160–0.001	0.0150–0.001	0.007

## Discussion

AC in brain PET/MRI studies is a problem that has swiftly evolved since the first PET/MRI scanners were installed. Many brain PET studies, static and dynamic, require a reference region to normalise the global activity distribution [35, 36]. While most AC-PET/MRI studies have been focused on static PET imaging experiments, it is not well understood how the non-uniform bias introduced by inaccurate PET AC can affect the outcome of dynamic PET analyses.

In this work, we evaluated the impact of different levels of accuracy in PET AC in a quantitative dynamic brain [^18^F]-FDOPA-PET/MRI study. In the present study, we used as reference an atlas-based μ-map, based on MRI-T1w images [9], whose reliability has been demonstrated in different independent studies [14, 15], consistently showing errors < 3% compared to CT in different brain regions. The evaluated μ-maps were the two μ-maps provided by the vendor with the software version VB20P, one based on the two-point Dixon MR sequence, and the other one including also bone, extracted from the dUTE MRI sequence. In addition, we included an in-house-developed MRI-based AC, already evaluated in previous studies encompassing static PET experiments [6].

While the μ-map_DXN_ does not contain bone information, the μ-map_dUTE_ represents the bone information with one constant LAC value (0.15 cm^−1^). In contrast, the μ-map_dUTE−R2_ and pCT contain the bone information in a continuous spectrum of LAC values, more similar to how CT represents the bone information. Independently of the LAC values, the level of overlapping of bone from μ-map_dUTE_ and μ-map_dUTE−R2_ compared to pCT was measured with the Jaccard index and NMI, resulting in the in-house-developed μ-map showing more overlapping.

We further calculated the E_n_ in absolute quantification at the different time points of the dynamic PET scan, by comparing each PET reconstructed frame using μ-map_DXN_, μ-map_dUTE_, and μ-map_dUTE−R2_ compared to pCT. In general, the activity concentration measured in PET-reconstructed images obtained with μ-map_DXN_, μ-map_dUTE_, and μ-map_dUTE−R2_ were lower than those obtained with pCT (E_n_ < 0%), indicating that bone (volume or density) in μ-map_dUTE_ and μ-map_dUTE−R2_ was underestimated compared to pCT. Results showed that the in-house-developed μ-map_dUTE−R2_ outperformed the two μ-maps provided by the vendor, resulting in an error of < 6%, below the test-retest error in [^18^F]-FDOPA-PET studies [30]. μ-map_dUTE_ led to more accurate PET images (lower E_n_) compared to μ-map_DXN_, as expected, due to the inclusion of bone in the μ-map. E_n_ measured along time was constant in all subjects with all the μ-maps after 8 min post-injection.

The non-uniform E_n_ across the brain volume was illustrated by the different mean E_n_ values obtained between the striatum, cerebellar cortex, and occipital pole (Table 2 and Fig. 3). Independent of the μ-map used for the PET reconstruction, the striatum showed the lowest E_n_, while the cerebellar cortex and occipital pole showed significantly higher E_n_, attributed to their proximity to the bone, as also observed in previous studies [37]. This was more clearly observed in the voxel-wise E_n_ maps calculated for each μ-map (Fig. 4), which showed the non-uniform E_n_ dependent on the proximity with the bone.

The measured *K*_*i*_ values with the μ-maps under evaluation were overestimated compared to the pCT (Tables 3 and 5, and Fig. 6), due to the higher differences between the central regions with respect to the outer regions of the brain, measured with μ-map_DXN_, μ-map_dUTE_, and μ-map_dUTE−R2_ than with pCT. As a result, higher ratios in both factors of the Patlak equation were obtained for the μ-maps under evaluation compared to pCT. This effect was attributed to the different non-uniformity observed in each voxel-wise E_n_ map, obtained with each μ-map.

This effect also explained why, using the cerebellar cortex as reference region in the Patlak analysis, PET-reconstructed images using μ-map_DXN_ resulted in more accurate *K*_*i*_^cer^ values compared to μ-map_dUTE_. However, using the occipital pole as reference region, PET-reconstructed images using μ-map_dUTE_ resulted in more accurate *K*_*i*_^op^ values compared to μ-map_DXN_. These results, even though initially unexpected, are explained by the different level of bias (E_n_) measured in the striatum with respect to the cerebellar cortex and occipital pole for each μ-map (Table 2).

In general, higher differences in *K*_*i*_ values between AC methods were observed when using the occipital pole as reference region (Fig. 6), which was attributed to the larger bias observed in the occipital pole as compared to the cerebellar cortex, with respect to the striatum (Fig. 4).

Using μ-map_dUTE−R2_ for PET-AC consistently produced the closest results to pCT, obtaining more accurate kinetic parameters using the cerebellar cortex as reference region, as compared to the occipital pole.

All the results presented in this work are subject to possible inaccuracies introduced by our reference, the pCT. On the other hand, the accuracy achieved by the pCT has previously been compared to CT [9, 14, 15, 21], demonstrating that the error lays below the test-retest variation among subjects in [^18^F]-FDOPA studies [30], and therefore, we considered it as a reliable reference.

After the last software upgrade of our Biograph mMR scanner from VB20P to E11, the vendor provided a new μ-map consisting of a combination of the Dixon μ-map, including bone extracted from a segmented skull from a patient MR image [38]. This approach showed errors of < 5% in most brain regions (in a static PET experiment) in the work presented by Koesters et al. Even though this approach showed closer results to CT as compared to the vendor-provided Dixon and dUTE μ-maps, this μ-map could not be included in the present study.

Regarding the clinical impact of the present study, we showed that all the μ-maps showed similar statistically significant differences between healthy controls and patients in the *K*_*i*_^cer^ values and no differences using the *K*_*i*_^op^ values. However, using pCT as μ-map, the *p* value was slightly smaller, followed by μ-map_DXN_, μ-map_dUTE−R2_, and μ-map_dUTE_. It is worth noticing that three patients were taking clozapine by the time the experiment was performed, which might reduce the dopamine synthesis capacity [27]. However, the findings from this study were not affected by removing these three patients from the analysis.

## Conclusions

Non-uniform AC in brain PET/MRI studies may significantly influence the outcome of PET data analysis, when a reference region is required for normalisation or kinetic modelling. We have shown that results are more accurate when the E_n_ in the region of interest (striatum in our case) and the reference region (cerebellar cortex or occipital pole in our case) are similar. The in-house-developed AC method, μ-map_dUTE−R2_, produced the most accurate results in PET absolute quantification and kinetic parameters using a pCT as reference. Out of the μ-maps from the vendor, μ-map_dUTE_ produced more accurate parametric information than μ-map_DXN_ when the occipital pole was used as reference region, while μ-map_DXN_ produced more accurate parametric information when the cerebellar cortex was used as reference region. This effect was attributed to the bias introduced by the bone contained in the cerebellar region in μ-map_dUTE_, increasing the relative differences in absolute quantification between the reference region and the region of interest, demonstrating the negative impact that inaccurate bone identification can potentially induce in quantification.

## Data Availability

The datasets used and/or analysed during the current study are available from the corresponding author on reasonable request.

## Abbreviations

2TCM: Two-tissue compartment model
AC: Attenuation correction
ANOVA: Analysis of variance
BP_ND_: Non-displaceable binding potential
CT: Computed tomography
dUTE: Dual ultrashort time echo
DXN: Dixon
FDG: Fluorodeoxyglucose
FDOPA: Fluorodopa
LAC: Linear attenuation coefficient
MNI: Montreal Neurological Institute
MPPF: Methoxyphenyl-(*N*-2′-pyridinyl)-p-fluoro-benzamidoethyipiperazine
MP-RAGE: Magnetization-prepared rapid gradient-echo
MRI: Magnetic resonance imaging
NMI: Normalised mutual information
pCT: Pseudo-CT
PET: Positron emission tomography
PiB: Pittsburgh compound B
SPM: Statistical parametric imaging
SPSS: Statistical Package for the Social Sciences
SRTM: Simplified reference tissue model
TAC: Time activity curve
TE: Time echo
UTE: Ultrashort time echo

## Footnotes

1
http://www.fil.ion.ucl.ac.uk/spm/

2
http://cmictig.cs.ucl.ac.uk/niftyweb/
